# Production Technologies and Application of Polymer Composites in Engineering: A Review

**DOI:** 10.3390/polym17162187

**Published:** 2025-08-09

**Authors:** Milan Bukvić, Saša Milojević, Sandra Gajević, Momčilo Đorđević, Blaža Stojanović

**Affiliations:** 1 Faculty of Engineering, University of Kragujevac, Sestre Janjić 6, 34000 Kragujevac, Serbia; milanbukvic76@gmail.com (M.B.); blaza@kg.ac.rs (B.S.); 2 Military Academy, University of Defence, Veljka Lukića Kurjaka 33, 11042 Belgrade, Serbia; djmoca71@gmail.com

**Keywords:** industry, materials and technology, polymer composites

## Abstract

Composite materials have been increasingly used in various branches of industry, transport, construction, and medicine—as well as in other sectors of the economy and science—in recent decades. A significant advancement in the improvement of composite material characteristics has been achieved through the use of nanoparticles, which substantially enhance the properties of the base material, whether it is the matrix or the reinforcing phase in hybrid composites. The broad application of polymers and polymer composites in many areas of engineering has had a significant impact on reducing friction and wear, improving the thermal characteristics of individual components and entire technical systems, enhancing electrical conductivity, reducing the specific weight of components, lowering noise and vibration levels, and ultimately decreasing fuel consumption, production costs, and the costs of operation and maintenance of technical systems. This paper explores the potential applications of polymer composites in various assemblies and components of conventional vehicles, as well as in hybrid and electric vehicles. Furthermore, their use in medicine and the defense industry is examined—fields in which some authors believe composites were first pioneered. Finally, aviation represents an indispensable domain for the application of such materials, presenting unique exploitation boundary conditions, including dynamic environmental changes such as variations in temperature, pressure, velocity, and direction, as well as the need for high levels of protection. Future research can be unequivocally focused on the structural and technological advancement of polymer composites, specifically through optimization aimed at reducing waste and lowering production costs.

## 1. Introduction

Constant demands for materials with increasingly superior mechanical, thermal, and electrical properties have led to the development of a wide range of new materials, all of which share a common characteristic: they are composed of two or more constituent materials and are therefore referred to as composite materials. These materials exhibit enhanced characteristics compared to their individual components, resulting in a synergistic effect.

Historically, the concept of composite materials dates back to ancient times. Early examples include mud bricks reinforced with straw by the Mesopotamian and Egyptians, as well as the use of multiple materials in crafting bows and arrows by the Mongols [1].

Today, the use of polymer composites is primarily associated with the production and application of plastics such as polyesters and polyvinyl, as well as various types of synthetic rubber [2]. The first commercial composites were developed for their improved strength and reduced weight. A significant shift in composite material development and mass production occurred during the Second World War [3]. The first major application of composites in the automotive industry was recorded in the USA in 1947 [2], and Japan subsequently became a global leader in the use of composite materials in this sector [4]. In the early 1990s, McLaren introduced the first carbon fiber car [5].

The key raw materials for producing composites are petroleum-based products, though carbon has become increasingly important across many industrial sectors and in daily life, displacing metals and their alloys in numerous applications. Concurrently, global trends toward sustainable technologies, ecological responsibility, and the need for extensive recycling have elevated the importance of composite materials even further [6].

Among other benefits, composites are used because of their low susceptibility to corrosion, the ease with which complex elements and assemblies can be designed, and their relatively low maintenance requirements [7].

The primary advantage of composites over traditional materials lies in their unique combination of strength, stiffness, and low weight. For manufacturers, it is crucial to determine the appropriate manufacturing technology, thermal treatment, and optimal ratios of matrix and reinforcement materials to achieve effective reinforcement [8]. On average, the tensile strength of composite materials is up to 15 times greater than that of conventional materials [9].

Composite materials also offer significant recycling potential—both due to the availability of effective recycling processes and a high degree of recyclability, as well as relatively low recycling costs [6]. Furthermore, total production costs are considered low in relation to the excellent performance and broad applicability of composites across various industries [10].

More recently, natural fiber composites have seen growing adoption, further improving environmental sustainability and, in some cases, reducing flammability [11,12]. One of the most important metrics in comparing composites to conventional materials is specific strength, defined as the ratio of strength to weight. For example, carbon fiber composites weigh only 25% of steel and up to 70% less than aluminum-based materials [13]. Additionally, multi-layer composites can absorb significantly more energy than conventional steels, enabling up to a 60% reduction in the weight of front-end vehicle components [14].

Polymer composites are increasingly utilized in the automotive industry due to their potential to reduce vehicle weight, which directly impacts fuel consumption and harmful gas emissions. The use of fiber-reinforced polymer matrices (carbon, glass, or natural fibers) enables a significant reduction in component mass while maintaining or even enhancing mechanical properties compared to metallic alternatives. Typical applications include bumpers, door panels, instrument dashboards, and chassis components, as highlighted by Shah et al. (2014) [15] and Jawaid and Abdul Khalil (2011) [16]. The development of thermoplastic composites has facilitated easier recycling and processing, making them an attractive solution for mass production in the automotive sector (Mohanty et al., 2000) [17]. According to Yang et al. (2020) [18], the combination of biodegradable polymers and natural fibers allows for the production of environmentally friendly automotive components, aligning with trends in sustainable mobility.

Polymer composites in the aerospace industry represent a key technology for achieving high structural efficiency with minimal weight, contributing significantly to fuel consumption reduction and increased payload capacity. Modern aircraft such as the Boeing 787 Dreamliner and Airbus A350 contain over 50% composite materials by weight, underscoring the importance of this technology in contemporary engineering. Fiber-reinforced polymers, especially carbon fiber-reinforced polymers (CFRP) with epoxy matrices, are used for wings, fuselages, vertical stabilizers, and engine components due to their high strength, stiffness, and fatigue resistance (Campbell, 2010) [19]. Techniques such as autoclave curing and additive manufacturing enhance component property precision and control, while reducing production costs and waste (Thomson et al., 2018) [20]. According to Gibson et al. (2021) [21], the integration of nanoparticles into aerospace composites further improves impact resistance, which is particularly critical for aircraft safety.

In the medical sector, polymer composites are increasingly employed for implants, orthopedic devices, dental restorations, and bio-materials that require high bio-compatibility and mechanical stability. Their adaptability, light weight properties, and tunable properties through nanophase modification make them exceptionally suitable for use in modern medicine. According to Kalita et al. (2007) [22], bio-active composites based on poly-lactic acid and hydroxyapatite exhibit excellent integration with bone tissue and support osteointegration. In dentistry, polymer composites are used for fillings, artificial crowns, and root-building materials due to their aesthetics and abrasion resistance, as demonstrated by Ferracane (2011) [23] and Ilie and Hickel (2015) [24]. Emerging trends include the use of nanocomposites that combine antibacterial properties with enhanced mechanical strength, which is particularly important in the prevention of infections in implantology.

The main contribution of this paper is a systematic review of modern polymer composite production methods, along with a comprehensive overview of their advantages and disadvantages in various fields of engineering. The paper primarily focuses on the key characteristics of polymer composites, such as strength, corrosion resistance, lightweight properties, and thermal stability. It also explores the use of composites in industries including automotive, aerospace, defense, and healthcare, comparing their performance with that of traditional materials like metal alloys, ceramics, plastics, and rubbers.

## 2. Polymer Composites: Characteristics and Manufacturing

Composites are hybrid materials composed of two or more constituent substances with significantly different physical and chemical properties. When combined, these materials form a composite with superior overall performance compared to each individual component. Importantly, the original materials remain physically and chemically distinct within the final structure—they do not dissolve or merge into a single phase—resulting in visible differentiation.

**Figure 1:**
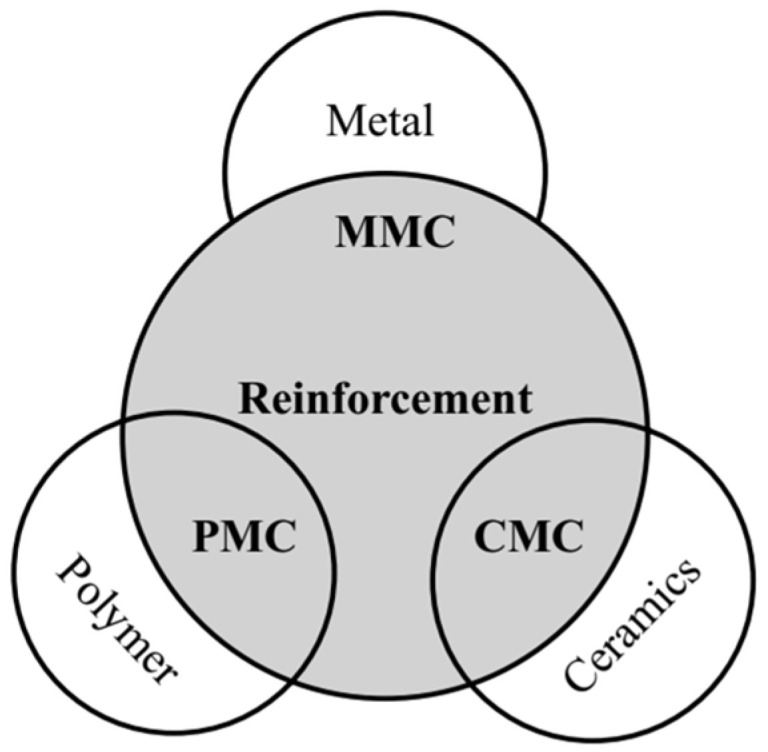
Types of composites [25].

A composite typically consists of a matrix phase, which constitutes the majority of the volume, and a reinforcement phase, which provides the desired strength and stiffness. Composites are generally classified based on the matrix material, which can be metal (metal matrix composites or MMCs), polymer (polymer matrix composites or PMCs), and ceramic (ceramic matrix composites or CMCs), as shown in (Figure 1). Among these, polymer matrix composites are the most widely used due to their low density, ease of processing, and cost-efficiency [25].

Among these, polymer matrix composites are the most widely used due to their low density, ease of processing, and cost-efficiency.

In PMCs, the polymer matrix is typically reinforced with fibers, such as carbon fibers, oxide fibers, and nanotube, which significantly enhance the mechanical and functional properties of the composite. In addition to reinforcement, additives and modifiers may be incorporated into the base polymer mixture to further improve work-ability, mechanical strength, thermal resistance, and service life [25,26].

Due to their superior performance and growing applications across various industries, a broad spectrum of technological processes has been developed for the manufacturing of polymer composites. These processes strongly influence key mechanical properties, including tensile strength, impact resistance, hardness, fatigue resistance, and corrosion resistance, as well as the economic feasibility of both production and application [16].

### 2.1. Conventional Manufacturing Technologies

Conventional manufacturing methods for polymer composites include the following:

- Extrusion molding;
- Injection molding;
- Calendaring;
- Hot pressing;
- Resin transfer molding;
- Filament;
- Winding;
- Pultrusion;
- Vacuum bagging.

These techniques ensure process repeatability and enable precise control over manufacturing parameters to consistently produce high-quality composites [27]. However, the complexity of part geometry and cross-sectional shape can significantly complicate design and increase tooling costs. This challenge has led to the development and adoption of advanced manufacturing techniques for polymer composites.

### 2.2. Advanced Manufacturing Technologies

Several modern techniques have been developed to address the limitations of traditional molding processes:

- Surface Coating Technology: This method involves applying a thin film onto a prepared substrate surface. Its key advantages include applicability to a wide range of materials, adaptability to various operating conditions, and low cost of raw materials and process execution. However, controlling coating thickness and the need for post-processing are common drawbacks. Applications include corrosion protection, pipeline construction, antibacterial coatings, and drug handling systems [28,29];
- Additive Manufacturing: Additive manufacturing operates on a bottom-up layering principle, allowing for high precision and the formation of stable internal structures [30,31]. The major advantages are design flexibility and ease of automation. However, limitations include the restricted volume of processed material and relatively slow production speeds [32,33]. Additive manufacturing is widely used in biomedicine [34,35], electronics, and aerospace applications [36,37];
- Magnetic Pulse Powder Compaction Technology: This technique consolidates powders using pulse-modulated electromagnetic field pressure. It is advantageous due to low cost, fast processing, and simplicity of use, but suffers from low overall efficiency. Its applications span medicine [38], ceramic composite manufacturing [39], and packaging materials [40].

### 2.3. Influence of Manufacturing Processes

The manufacturing or processing technique is one of the key factors affecting the mechanical performance and long-term durability of polymer composites. The selection of an appropriate technique depends on the following:

- The type and characteristics of the matrix;
- The form and geometry of raw materials;
- The desired final shape of the composite;
- The targeted application, whether for macro or micro use environments [41].

Conventional manufacturing processes for polymer composites encompass a set of techniques that enable the efficient integration of a polymer matrix with reinforcing materials such as fibers or fillers, aiming to produce materials with enhanced mechanical properties and durability. One of the most widespread methods is hand lay-up, which involves the layered placement of reinforcing fibers into a mold, followed by the application of polymer resin onto them. This process is relatively simple and accessible, making it suitable for small batch production and prototyping; however, it requires careful operator attention to ensure thorough fiber impregnation and minimize air bubble entrapment in the material.

Vacuum infusion represents a more advanced technique in which dry fiber layers are arranged within a mold, and the polymer matrix is introduced under vacuum pressure, resulting in better fiber impregnation and reduced defects such as air bubbles. This process provides higher quality and more consistent results compared to hand lay-up and is frequently employed in aerospace and automotive industries where composite mechanical performance is critical.

For thermoplastic composite manufacturing, methods such as extrusion and calendaring enable the mixing of a thermoplastic matrix with reinforcing fibers, after which the material is shaped into tapes or sheets ready for further processing. These techniques allow rapid production and recyclability but require precise control of parameters such as temperature and cooling rate to ensure composite homogeneity and stability.

Traditional technologies like compression molding and autoclave curing are used to produce high-quality components, particularly in sectors such as aerospace. Compression molding involves applying high pressure and temperature to shape and cure the composite, while autoclave curing adds additional pressure within a controlled environment, leading to maximum material density and mechanical performance. These methods are more expensive and require complex equipment but deliver products of superior quality.

Overall, the choice of conventional polymer composite manufacturing processes depends on multiple factors including production volume, desired end-product properties, economic feasibility, and application specifics. Each method offers a compromise among quality, cost, and time efficiency, which is crucial for successful industrial implementation.

### 2.4. Macro-, Micro-, and Mesoscale Considerations

**Figure 2:**
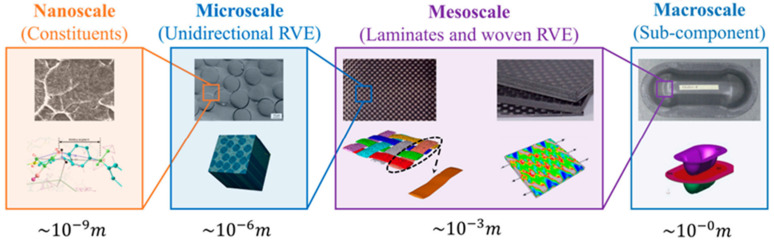
Schematic representation of polymer composites in different scales with the application of computer modelling [42].

Historically, both macro- and microscale approaches have been widely used in composite material design. However, the recent surge in the use of nanomaterials (e.g., nanofibers, nanoparticles) as reinforcements has brought significant attention to the nanoscale. Nevertheless, for a comprehensive understanding of material evolution, wear mechanisms, and damage progression, the mesoscale has emerged as a crucial level of analysis (Figure 2) [42].

The influence of reinforcement size on the properties of polymer composites is a crucial factor that significantly determines the mechanical, thermal, and functional characteristics of the final material. Smaller dimensions of reinforcing particles or fibers, particularly at the nanoscale, lead to an increased specific surface area of contact between the reinforcement and the polymer matrix, resulting in improved adhesion and more efficient load transfer. This is manifested through a significant enhancement in strength, elastic modulus, and fatigue resistance of the material. On the other hand, an increase in reinforcement size can lead to stress concentration and defect formation, which negatively affects the integrity and homogeneity of the composite.

Additionally, the size of the reinforcement influences the composite’s microstructure, including the distribution and orientation of fibers or particles, which directly determines the anisotropy of mechanical properties. Nanoscale reinforcements enable better space filling within the matrix, reducing microcracks and increasing resistance to defect propagation. However, the reduction in reinforcement size is often accompanied by challenges related to dispersion and agglomeration, which can adversely affect performance if not properly controlled.

In summary, optimizing the size of the reinforcement represents a balance between macroscopic and nanoscale properties, aiming to achieve maximum synergy between the matrix and the reinforcement, thereby maximizing the mechanical performance, durability, and functionality of polymer composites.

A special class of polymer composites is represented by those reinforced with natural biodegradable fibers. Several researchers have investigated the processing techniques and hybridization methods for producing hybrid bio-composites, which combine natural fibers with synthetic reinforcements or multiple types of natural fibers to achieve improved mechanical and thermal performance. The goal is to optimize the trade-off between sustainability and engineering performance. In addition to reducing the environmental footprint, these materials often offer advantages such as low density, good acoustic damping, and cost-effectiveness. However, they also face certain limitations, including moisture absorption, poor interfacial adhesion, and variability in fiber properties, which can affect consistency in performance [43,44,45].

## 3. Polymer Surface Treatment Technologies

Surface treatment technologies for polymers and polymer composites play a critical role in enhancing inter-facial adhesion, mechanical performance, corrosion resistance, and functional compatibility with other materials. These treatments are particularly important when reinforcing polymers with fibers (e.g., glass, carbon, or natural fibers), as strong inter-facial bonding between the matrix and reinforcement is essential for optimal load transfer and durability.

Surface treating methods can be broadly classified into chemical, physical, and physical-chemical categories [46,47,48,49,50].

As explained above, some of the most modern and advanced technologies for producing polymer composites—such as surface coating technology [28], additive manufacturing [19,20,21,22], and magnetic pulse powder compaction [33]—are accounting for an increasingly significant share of total composite production.

### 3.1. Surface Coating

Surface coating technology involves the formation of a thin film layer on the surface of a substrate, significantly enhancing the surface properties of the underlying material. Its main advantages include the ability to apply a wide range of materials, adaptability to various working environments, and high economic efficiency. However, key disadvantages include the difficulty of precisely controlling film thickness and the frequent requirement for post-treatment of the surface.

Polymer composites produced using surface coating technology have found applications in areas such as drug delivery systems, corrosion protection, antibacterial surface coatings, pipeline protection, and micro-battery production [34].

In general, surface coating is a technique used to enhance the functional characteristics of a material by depositing a thin surface layer, which may be polymeric or non-polymeric. When applied to polymer substrates, these coatings yield composite materials widely used in bio-medicine. In contrast, coatings applied to non-polymeric substrates—such as metals or ceramics—result in composites primarily used for anti-corrosion protection in aggressive chemical or environmental conditions [51].

The surface coating technology includes several specialized deposition methods, such as the following:

- Plasma sputtering [51];
- Magnetron sputtering [52];
- Electrochemical deposition [53];
- Sol–gel processing [54].

Among these, plasma spraying is one of the most widely used techniques in biomedical applications [55]. In this process, a plasma arc heats powder particles to temperatures of several thousand degrees Celsius, melting or semi-melting them. The molten particles are then accelerated toward the substrate using an injector and deposited at high velocity, forming a dense and adherent surface film. The resulting coatings exhibit excellent wear resistance, corrosion resistance, high-temperature stability, and good thermal insulation properties.

**Figure 3:**
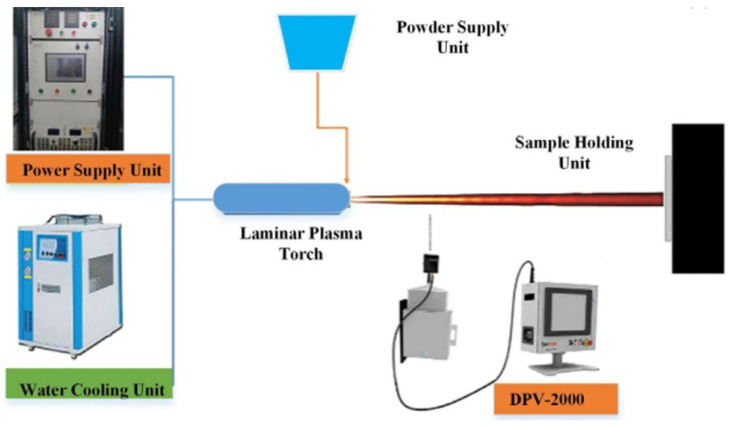
Schematic representation of plasma spraying technology [56,57].

A schematic representation of this composite surface-coating process is provided in (Figure 3) [56,57].

Under the influence of the plasma jet—specifically its kinetic and thermal effects—the powder is melted and dispersed onto the substrate. Upon impact, molten particles form a film on the substrate surface [56]. The surface roughness of the substrate directly influences the bond strength between the film and the substrate. Higher roughness typically results in stronger adhesion [58]. Additionally, direct contact between particle droplets and the substrate’s convex peaks reduces heat dissipation, maintaining an elevated temperature for a longer period. This condition facilitates the formation of chemical bonds between powder particles and the substrate surface [57].

The physical properties of plasma-sprayed coatings—such as porosity, bonding strength, coating thickness, hardness, electrical conductivity, and thermal conductivity—have been extensively investigated [57]. It has been demonstrated that spray velocity significantly affects coating porosity. Specifically, increased spray speed results in reduced porosity. Furthermore, the intrinsic properties of the coating material also influence porosity and adhesion strength. Among all parameters, coating thickness exerts the most critical influence on performance. With increasing thickness, thermal conductivity decreases, internal stress accumulates, and the formation of cracks is more likely. Simultaneously, the mechanical strength, hardness, and electrical conductivity decline. Therefore, coatings should be applied as thinly as possible, while ensuring that their essential functional properties are preserved [58].

**Figure 4:**
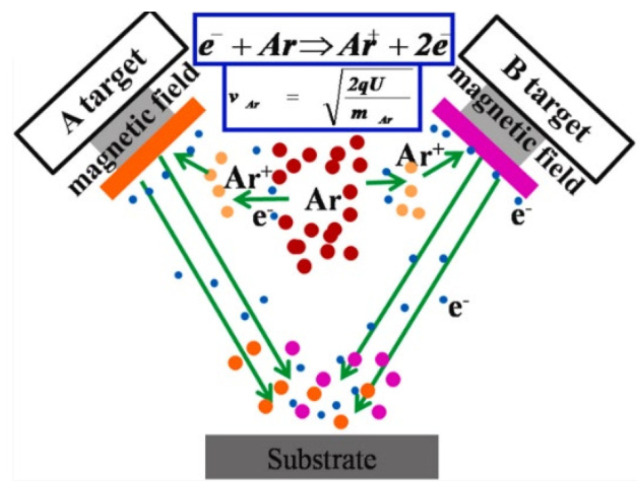
Schematic representation of the magnetic sputtering process [61].

Magnetron sputtering involves the bombardment of a target by energetic particles. During this process, the incident particle transfers momentum to the target atoms, which then propagate this impulse to neighboring atoms. This initiates a cascade or “domino effect,” as illustrated in Figure 4 [59,60]. A film growth and adhesion mechanism in this context is complex, and no universally accepted theory currently exists to fully explain these phenomena [61].

High-power pulsed magnetron sputtering (HiPIMS) has recently been developed as an advanced coating technique. This method combines the high-energy deposition benefits of pulsed discharges with conventional magnetron sputtering, resulting in faster deposition rates. Films produced by HiPIMS exhibit higher density [62], improved optical characteristics [63], enhanced adhesion, and increased toughness of the resulting composite [64]. These materials are primarily used in biomedical applications.

Electrochemical deposition is a technique used to obtain polymer composites at low to moderate temperatures, in contrast to the previously mentioned methods, which operate at high temperatures [65].

**Figure 5:**
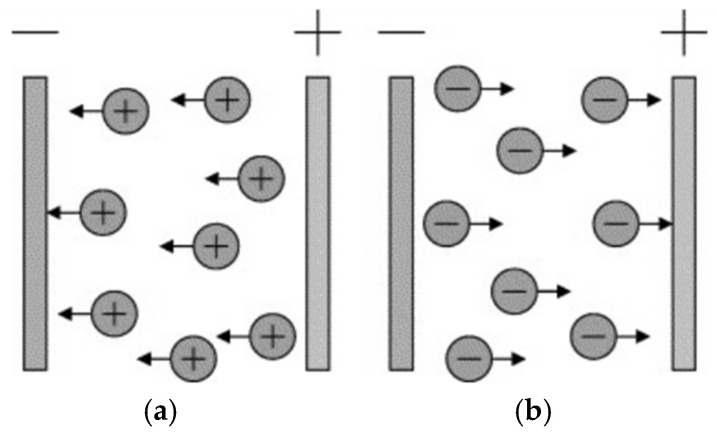
Electrochemical deposition or EDP electrophoresis: (**a**) cathodic EDP; (**b**) anodic EDP [61].

The principle of electrochemical deposition, also known as electrophoretic deposition, is illustrated in Figure 5 [66].

Electrophoresis involves the movement of charged particles in suspension toward an oppositely charged substrate under the influence of a strong electric field, where the particles deposit onto the surface of the conductive substrate, forming a uniform layer. In recent years, this technique has been increasingly applied to the fabrication of composite materials for biomedical applications [28,67].

Among its advantages, electrochemical deposition offers greater flexibility than plasma spraying and magnetron sputtering, as it enables precise control of film thickness by adjusting parameters such as deposition time, applied voltage, and particle concentration in the solution [68,69]. Furthermore, the operating temperature of electrophoretic deposition is significantly lower than that of plasma spraying and magnetron sputtering, which helps prevent the generation of thermal stress during the deposition process and allows for coating of substrates with complex geometries [68].

**Figure 6:**
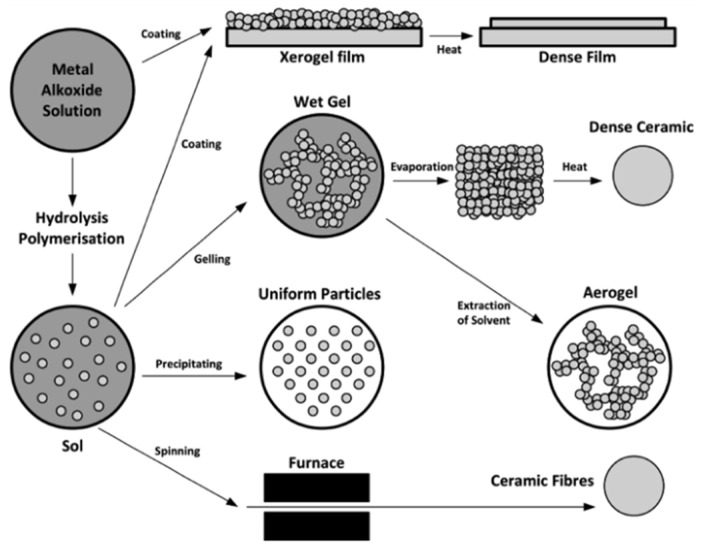
Schematic representation of sol–gel technology [28].

Sol–gel technology is an advanced method for producing composite materials, particularly for the preparation of thin oxide films [70]. The underlying principle of this technique is illustrated in Figure 6.

Prior to coating application, surface pre-treatment—namely, substrate cleaning—is performed, as the condition of the substrate surface significantly influences the structure and physical properties of the resulting film [70]. Most polymer resins exhibit low surface free energy and a lack of polar functional groups, which leads to weak adhesion [71]. Therefore, it is essential to pre-treat the substrate surface to enhance adhesion between the film and the polymer substrate, using techniques such as ion functionalization [72] and chemical abrasion [73].

The second step in the sol–gel process is film deposition. Sol–gel techniques can generally be categorized into two types: sol–gel dipping and sol–gel spin coating [61]. In both cases, the wet gel must be dried and sintered before the final film is formed. However, due to the high liquid content of the wet gel, significant shrinkage and cracking can occur during the drying phase. Therefore, careful control of film thickness is essential.

Plasma spraying and magnetron sputtering typically require vacuum environments due to their high operating temperatures and the susceptibility of coating materials to oxidation. In contrast, sol–gel processing does not require a vacuum, as it operates at lower temperatures. Nevertheless, it often results in film cracking during drying, and the overall preparation time is relatively long [61].

### 3.2. Additive Production Technology

**Figure 7:**
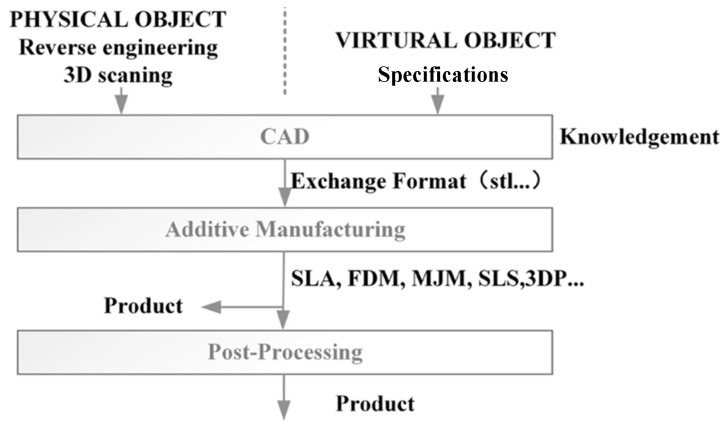
Schematic representation of three-dimensional printing [61].

Additive manufacturing employs a “bottom-up” approach, in which materials are deposited layer by layer, offering advantages such as the formation of complex material networks and the simplification of technological processes. However, typical drawbacks of this method include limited material options and relatively slow production speeds. In practice, additive manufacturing is also referred to as rapid prototyping or 3D printing, as it is a multidimensional process that builds structures sequentially by stacking layers of material [74]. Three-dimensional printing is a prototyping technique that utilizes powdered metals, polymers, and binding agents to fabricate objects layer by layer, as illustrated schematically in Figure 7 [75].

Unlike conventional surface coating techniques, three-dimensional (3D) printing is not a standalone technology, as it integrates multiple disciplines, including computer technology, computer-aided design (CAD), laser systems, printing, and computer numerical control (CNC) machining [76]. The field of 3D printing has evolved rapidly, giving rise to various methods such as fused deposition modeling (FDM) [77], selective laser sintering (SLS) [78], powder-based and inkjet 3D printing (3DP) [79], and stereo lithography (SLA) [80].

This technology enables the printing of thermoplastic polymer materials [81,82,83]; however, most of the polymer materials currently used in such composites exhibit relatively low mechanical performance [84].

### 3.3. Magnetic Technology

Pulsed powder compaction using magnetic technology is a method for producing polymer composites in which powders are compacted under pressure generated by a pulsed, modulated electromagnetic field. This approach offers several advantages, including high cost-efficiency, rapid processing, and relatively simple technological implementation. However, it also has limitations—it is generally suitable only for simple part geometries and is associated with significant energy losses. The main application areas of composites produced by this method include biomedicine [38], ceramic components [39], and various packaging materials [40].

The quality of the powder mold is primarily determined by the compaction density. Conventional quasi-static methods—such as extrusion molding, injection molding, and hot press casting—tend to result in non-uniform density distribution within the compacted material. Friction between powder particles and the die walls leads to energy loss and generates significant residual stress during sintering, often resulting in cracking of the final part.

**Figure 8:**
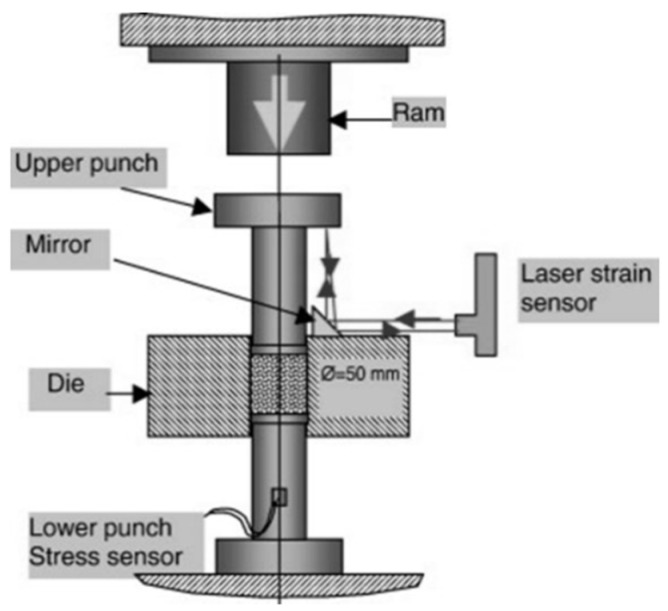
Schematic representation of high-speed compaction [61].

In contrast, magnetic pulsed powder compaction is a dynamic, high-speed process suitable for rapid prototyping. Similar to other high-velocity compaction techniques—such as explosive compaction—it relies on stress wave propagation to achieve densification, as schematically illustrated in Figure 8 [85,86,87]. Researchers have investigated the influence of various compaction speeds, which are key factors in determining product quality. One of the ongoing technical challenges has been the control of explosive compaction speed [77]. By adjusting the release height of the impacting hammer, it is possible to regulate compaction velocity; however, this also results in increased noise levels [88].

During the powder compaction process, powder particles undergo three successive stages: particle rearrangement, elastoplastic deformation, and fracture. Significant deformation generates substantial heat at the particle surfaces, resulting in localized welding. In magnetic pulse powder compaction, this process occurs extremely rapidly—within a few milliseconds or even microseconds—leading to local particle bonding through thermal effects [89].

Current research on magnetic pulse powder compaction technology primarily focuses on metal matrix composites [90,91,92,93,94], inorganic non-metallic materials [95,96,97], and other powders with favorable electrical and thermal conductivity.

This method offers several advantages, including high compaction density and uniformity, good mechanical performance, low production cost, and high throughput. However, the energy efficiency of the equipment remains extremely low. A significant portion of the input energy is absorbed by the magnetic field penetrating the work-piece; additional losses occur due to coil resistance heating and residual magnetic energy dissipation as heat. Although some energy loss can be mitigated by reducing the magnetic field penetration depth and using low-resistance coil winding, the overall energy efficiency remains below expectations. Consequently, improving energy recovery and overall efficiency continues to be a major challenge in this field [61].

## 4. Applications of Polymer Composite Materials

Polymer composites have found applications across diverse fields, primarily in the manufacturing of components and parts for transportation vehicles, medical devices, industrial equipment, and machinery designed to operate under extreme conditions.

### 4.1. Application of Polymer Composites in the Automotive Industry

The use of polymer composites in the automotive industry has seen a significant increase in recent years, complementing the traditional application of metal matrix composites. Metal composites consist of reinforcement fibers such as carbon, glass, or silver threads woven into metal matrices [98]. Due to their unique properties, metal matrix composites are widely employed in the automotive sector, primarily because of their advantages over conventional materials like steel and aluminum [99]. These composites are commonly used in the manufacture of brake pads, hoods, chassis, leaf springs, doors, sunroofs, engine mounts, bumpers, and other structural vehicle components [100].

Polymer composites in the automotive industry are manufactured from thermoplastic or thermoset polymers reinforced with materials such as carbon, glass, or aramid fibers. Prior to fabrication, the fibers are prepared and treated to enhance adhesion with the matrix. Molding processes include resin transfer molding, injection molding of thermoplastic composites with long or short fibers, pultrusion, and prepreg technology for high-performance parts. After molding, the components undergo thermal treatment, machining, and final painting. Quality control involves ultrasonic and X-ray inspections, as well as mechanical testing. These composites are utilized in body components, interior parts, structural elements, and performance vehicle components.

Brake pads are a critical component of a vehicle’s braking system. The materials used for brake pads must demonstrate reliable friction and wear performance under specified loads, speeds, and temperatures within defined operational ranges [101]. Phenolic composites are commonly employed in automotive brake pads due to their high coefficient of friction and reduced wear, both at room temperature and elevated temperatures up to approximately 250 °C [102].

Polymer composites are increasingly replacing traditional materials such as cast iron, steel, and aluminum in the production of brake linings [102]. Compared to conventional materials, brake pads made from composite materials are lighter, contributing to overall vehicle weight reduction and improved handling [103]. Additionally, composite materials generate significantly less brake dust and produce lower noise levels during braking [101].

**Figure 9:**
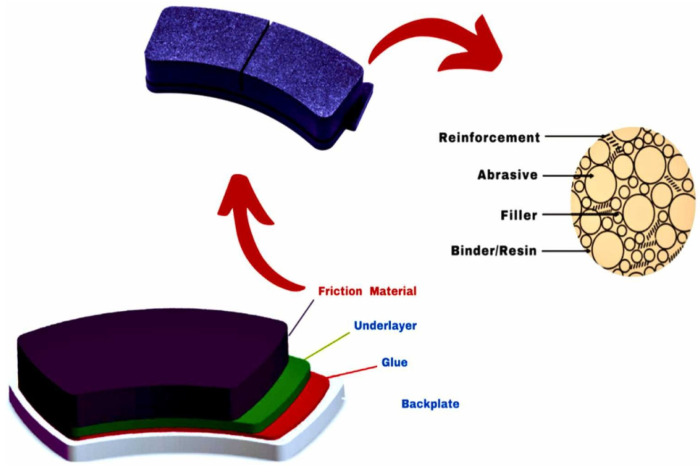
The brake pad structure is made of polymer composite materials [104].

A model of a brake pad made from polymer composite materials is shown in the Figure 9. The bottom layer, located between the friction material and the backing plate, acts as an adhesive that secures the friction material to the other layers. Four types of friction materials are identified: (1) reinforcement, (2) abrasive layer, (3) filling layer, and (4) binding material [104].

Polymer composites exhibit excellent strength and impact resistance while maintaining low weight. Considering that vehicle hoods are increasingly made from polymer composites, these materials provide exceptional crashworthiness, thereby enhancing occupant safety in the event of a collision [105].

Composite materials offer automakers greater design flexibility for vehicle hoods, enabling more complex shapes, forms, and opening/closing mechanisms [1]. Polymer composite hoods contribute to improved vehicle acceleration and handling by reducing overall weight, increasing the power-to-weight ratio, lowering noise and vibration levels, and maintaining stable temperatures under the hood [106].

Several studies [107] have demonstrated that composite materials can reduce hood weight by nearly 30% while preserving equivalent strength and resistance to torsional and bending stresses.

Composite materials are increasingly utilized in the production of vehicle chassis, which serve as the structural foundation of the vehicle and play a crucial role in improving performance and operational efficiency [108]. Among these, syntactic foam is gaining prominence as a core material in modern automotive designs, particularly for enhancing the stiffness of thin-sheet sandwich constructions.

Similar to their application in hood manufacturing, polymer composite materials are also employed in chassis fabrication, contributing to overall vehicle weight reduction, improved handling, and enhanced fuel efficiency [109]. Additionally, polymer composites possess intrinsic damping properties that reduce noise and vibration transmission through the chassis structure [110].

The use of these materials provides excellent fatigue resistance, allowing the chassis to maintain its mechanical performance and structural integrity over extended service life [111]. Numerous studies have confirmed that the integration of composite materials positively impacts vehicle performance—primarily by reducing mass, lowering fuel consumption, increasing power-to-weight ratio, and enhancing damage resistance [112,113].

An essential component of vehicle suspension systems is the leaf spring, which can be manufactured from glass fiber-reinforced epoxy polymer composites. In terms of fatigue resistance, such composite leaf springs demonstrate up to five times higher performance compared to their steel counterparts [114]. In addition, composite leaf springs offer a quieter and more comfortable ride, along with faster response to road irregularities, including bumps and vibrations.

Furthermore, composite leaf springs exhibit superior corrosion resistance and a lower likelihood of breakage, cracking, or permanent deformation [115]. They also provide significantly higher load-bearing capacity than steel springs and contribute to the development of so-called “silent suspension” systems in vehicles [111,116].

Another critical element of passive vehicle safety is the bumper, which is designed to absorb and dissipate energy during low-speed collisions, thereby minimizing damage to both the vehicle and its occupants [117].

Carbon and glass fiber-reinforced polymer composites, known for their excellent impact absorption properties, are widely used in automotive bumpers [118]. These composite bumpers significantly reduce vehicle damage and improve occupant safety by effectively absorbing and dissipating energy during collisions [119]. Additionally, they are generally easier and less costly to repair than conventional steel bumpers, particularly in cases of minor damage [120].

Composite materials also offer high stiffness and strength, enhancing the structural integrity and impact resistance of car doors. Compared to metals, they exhibit superior resistance to corrosion, wear, and fatigue [121]. In the event of a collision, doors made from polymer composites provide a higher level of protection for vehicle occupants. Moreover, they allow manufacturers to design doors with more complex geometries and integrated features [122].

Polymer composites also improve cabin comfort by offering superior acoustic insulation. Their specialized sound-dampening properties reduce noise transmission through the door panels [123]. For instance, carbon fiber-reinforced epoxy composites can absorb more deformation energy than steel while reducing component weight by up to 65% [124].

Composite materials are increasingly being used in the construction of vehicle roofs, offering multiple advantages such as improved natural lighting, enhanced ventilation, greater aesthetic appeal, and better vehicle acceleration [125]. However, the primary motivation for incorporating polymer composites into roof structures is weight reduction, which directly contributes to increased overall vehicle efficiency [126].

Vehicle roofs may be partially or fully glazed. In such cases, laminated composite glass—composed of multiple glass layers bonded with an interlayer of polycarbonate or polyvinyl—offers superior impact resistance and lower weight compared to standard or even reinforced glass options [127].

Continuous carbon fiber composites further enhance the performance of transparent sunroofs. In the event of partial or complete structural failure, these materials prevent glass fragmentation, thereby minimizing the risk of injury to vehicle occupants [128].

The application of composite materials in the manufacturing of internal combustion (IC) engine supports, housings, and vehicle body components is of particular importance [129]. Owing to their inherent thermal insulation properties, composite materials improve thermal management by reducing heat transfer from the engine to adjacent vehicle systems [130].

Crash safety can also be enhanced through the use of composite engine mounts, which are capable of absorbing and dissipating impact energy during collisions. Additionally, the use of composites in the engine bay facilitates the integration of surrounding systems, such as suspension components and mounting points, thereby enabling more efficient and innovative vehicle design [131].

Composite materials—formed by combining two or more constituents to achieve enhanced properties—are increasingly used in the production of vehicle fenders [132]. Thermoplastic composites, which can be reshaped through heating and cooling, are becoming particularly widespread due to their affordability and ease of processing [133]. Compared to thermoset composites, thermoplastics exhibit greater toughness, hardness, and stiffness, along with superior recyclability.

Fenders made from thermoplastic composites are lightweight, highly durable, and resistant to scratches, minor impacts, and collisions [134]. Moreover, repairing composite fenders is considerably simpler and more cost-effective than repairing metal fenders, as minor damage can typically be resolved through patching or adhesive bonding techniques [135,136].

The use of polymer composites in the production of vehicle cabin interiors, cockpits, auxiliary assemblies, and various components—traditionally made from plastics, rubber, thermoplastic sheets, and glass—is becoming increasingly widespread. Polymer composites enable the fabrication of entire assemblies or specific segments that are subjected to higher mechanical loads, significant temperature variations, or rapid changes in operating conditions.

Typical components manufactured from polymer composites include steering wheels, gear levers, cockpit paneling, interior and exterior mirrors, air diffusers for ventilation systems, windshield wipers, foot control pedals, and other auxiliary equipment [136].

Polymer composites based on PBO fibers represent a highly promising material for application in the automotive industry due to their exceptional strength-to-weight ratio and high thermal stability. The inert surface of PBO fibers hinders their integration with polymer matrices, but modern surface modification techniques—including silanization, ionic coordination with La^3+^ ions, plasma treatments, and nano-functionalization with CNT and GO—significantly improve interfacial adhesion. These treatments enable the formation of composites with enhanced interlaminar strength, modified tribological characteristics, and stable mechanical properties at elevated temperatures. In automotive components subjected to high mechanical and thermal loads—such as structural parts, braking system components, and thermal shields—modified PBO composites offer superior performance compared to conventional aramid or carbon fiber composites. The significantly reduced wear and lower coefficient of friction make them suitable for parts exposed to dynamic loading. Thanks to their exceptional heat resistance, they can be applied near engines or exhaust systems without property degradation. Their application contributes to reducing the overall vehicle weight, which directly impacts fuel efficiency and emission reduction. The effects of surface modification are long-lasting and maintained even under elevated temperatures and loading conditions, ensuring reliability in real-world operating environments. Engineering implementation of these composites requires further optimization of processing methods and compatibility with selected matrices. The integration of PBO composites represents a strategic step toward the development of lighter, more efficient, and more durable vehicles [137].

Beyond the applications mentioned above, composite materials are increasingly being adopted in advanced vehicle models, particularly those operating under demanding conditions—such as extreme speeds, heavy loads, harsh environmental influences, and variable operational regimes.

One especially important aspect is the improved recyclability of composite materials, which enables significant waste reduction and facilitates the reuse of previously utilized components in the production of new vehicles.

### 4.2. Application of Polymer Composites in Medicine

Fiber-reinforced composite materials, owing to their exceptional strength and biocompatibility, have found extensive applications in dentistry and orthopedics. Notably, significant technological advancements have been achieved in orthopedics, particularly in the fabrication of sports prostheses for the lower limbs [136].

Various aramid composite fibers are widely used in biomedical applications, including immobilization of body extremities, medical implants, and devices—especially within modern orthopedic medicine addressing severe injuries.

Polyamide (PA), commonly known as nylon, is a synthetic polymer valued for its high mechanical strength and is frequently utilized in implant manufacturing. Meanwhile, fibrous composites play a critical role in the production of prostheses and suture materials [138,139,140,141,142].

Bio-stable glass composite fibers demonstrate excellent load-bearing capacity in implants and possess antimicrobial properties due to the dissolution of bioactive glass particles that promote bone bonding [143].

Tissue engineering has seen intensive development recently, with collagen composites showing great promise in the reconstruction of damaged tissues. Fibrous composites composed of synthetic biodegradable polymers such as PLGA (poly(lactic-co-glycolic) acid), PLG, gelatin, and elastin (PGE) support dense cell proliferation and enable the maintenance of high cell densities.

A notable example of the growing use of composites in this field is the polyurethane (PU) heart patch reinforced with nickel oxide (NiO), fabricated via electrospinning. The PU/NiO nanocomposite significantly enhances delayed blood clotting, a critical process in cardiac tissue repair [144].

Wound healing is a critical area of application for polymer composites, particularly fibrin–collagen filament composites, which exhibit exceptional resilience to repeated compressions, thereby enhancing the elastic interaction between collagen and fibrin fibers. Fibrin actively participates in hemostasis and tissue repair, while a matrix gel based on collagen, gelatin, or elastin forms the composite’s structural foundation [145,146,147].

Biopolymers such as polylactic acid (PLA), polyglycolic acid (PGA), poly (lactic-co-glycolic acid) (PLGA), polycaprolactones (PCL), and polyester amides (PEA) find extensive use in biomedical applications including wound closure, postpartum recovery, tissue engineering, ligament, tendon and bone fixation, dentistry, and surgical implants [122].

The use of metal implants often raises issues such as allergic reactions caused by ionic interactions and the mismatch in elastic stiffness between the metal implant and the host tissue. Material fatigue also presents a significant challenge for metal implants. These problems can be largely mitigated by employing polymer composites, whose stiffness can be tailored to match that of the target tissue, thus reducing adverse reactions due to stiffness mismatch [148,149].

A wide variety of fiber-reinforced polymer composites are currently utilized in prosthetics, bone defect correction, and the manufacture of prostheses and sutures [150].

Composite fibers have demonstrated significant potential for both internal and external fixation of fractured human bones when combined with calcium phosphate and hydroxyapatite. Composites based on fundamental biopolymer matrices are primarily employed in the development of prostheses for the lower limbs, though there is growing potential for their application in upper limb prosthetics as well [151].

Both natural and synthetic degradable materials are widely used as scaffolds for bone repair and regeneration, owing to their excellent mechanical and biological properties [152]. The use of these materials in biomedical applications aligns with principles of environmental protection and sustainable development, and they are extensively utilized in the production of hydroxyapatite powders [153,154].

The skin, as the body’s largest organ, functions as the primary defensive barrier of the immune system. Due to its constant exposure to pathogens and various forms of damage, conditions such as infections, burns, and cuts frequently occur. The natural regenerative capacity of the skin can be impaired by such injuries but can be enhanced through the application of biodegradable and biocompatible polymer composites.

Polymer composites facilitate rapid transdermal drug delivery, accelerating wound healing and promoting drug penetration into muscle fibers and the skeletal system, thereby improving therapeutic outcomes. Polymer-based hydrogels serve as effective carriers for drug molecules, including anticancer agents, antibiotics, and antifungals.

Additionally, polymeric materials are widely used in the fabrication of wound dressings to promote healing, as well as in tissue engineering, which supports the regeneration of damaged or lost tissues primarily by stimulating new cell growth [155].

The advantageous properties of polymer composite materials—such as high strength and biocompatibility—underpin their extensive applications in dentistry and orthopedics. They offer an excellent alternative to traditional metal implants and devices, particularly in patients exhibiting metal allergies triggered by ionic reactions. Moreover, polymer composites provide superior elasticity and adaptability to the targeted anatomical sites and exhibit reduced material fatigue compared to metal implants. Polymer composites also possess certain disadvantages. Their resistance to UV radiation and weathering is limited, which can lead to degradation and a reduction in mechanical properties. They are sensitive to moisture and chemicals, affecting the adhesion and structural integrity of the composites. Manufacturing is complex and requires precise process control, which increases costs. Additionally, they exhibit lower resistance to high temperatures compared to metals, and recycling is challenging and often economically unfeasible. The stiffness of polymer composites may be lower than that of metals, limiting their use in structures that demand high resistance to bending and impact. These factors are critical considerations when selecting composites for engineering applications. [148,149].

Although the application of PBO fibers in medicine has been less prevalent, it is gaining increasing significance due to their biocompatibility, chemical stability, and high mechanical durability. The most important areas of application include surgical sutures, vascular implants, and reinforcement of orthopedic implants. A key challenge in this field is the control of biological interactions at the interface between the composite fiber and living tissue, necessitating modifications that enhance hydrophilicity and protein adsorption. Low-temperature plasma treatments enable the introduction of hydrophilic functional groups without damaging the fibers, resulting in improved cell adhesion and tissue regeneration. Chemical modifications, such as treatments with NaOH or methanesulfonic acid, increase the O/C and N/C ratios on the fiber surface, thereby improving bonding with bioactive matrices. Studies also demonstrate that interface-enhanced PBO composites exhibit reduced inflammatory response and improved long-term stability in implants. The transition from adhesive to cohesive failure modes in implants indicates greater reliability under biological conditions. This opens avenues for application in advanced areas such as smart prosthetics and tissue engineering scaffolds [137].

### 4.3. Application of Polymer Composites in the Military Industry

The military industry was among the first sectors to adopt polymer nanocomposite materials, as evidenced by numerous studies encompassing laboratory experiments, field trials, and real-world applications in military exercises and combat operations. The use of nanocomposites in the defense sector has grown remarkably, owing to their exceptional properties that provide enhanced protection for combat systems, vehicle crews, aircraft, vessels, and personnel operating in diverse combat environments.

Furthermore, these materials significantly improve the comfort of military personnel during operations, thereby increasing their survivability on the battlefield [156].

The widespread adoption of composites spans various military and defense applications, including optoelectronic and reconnaissance systems, military medicine, protective gear, textiles, management of automatic power supply systems, firearms, cold and special weapons, as well as enhancements in aircraft aerodynamics [157].

Armies worldwide employ a diverse range of vehicles, aircraft, ships, and drones for various operations, which imposes specific requirements distinct from those of civilian vehicles and aircraft. These requirements include fire resistance and retardation, high-temperature tolerance, electromagnetic shielding, cladding and armored for vehicle protection, enhanced ballistic performance, improved sensor capabilities, safety features such as shock, noise, and vibration absorption, and electrical energy storage [158].

In military shipbuilding, some of these requirements are met through the use of carbon-reinforced vinyl ester resin and phenolic laminate fiberglass plates, with the deck and roof constructed from individual composite elements. Polymer composites are also utilized in the manufacture of antennas, masts, and radar systems. For example, certain fighter jets, such as the American F-35, feature fuselage, wings, and horizontal and vertical stabilizers made of carbon fiber-reinforced composite polymers to enhance toughness, elasticity, and durability [159].

Polymer composites are highly suitable for producing various ballistic protective equipment, including armored plates, reinforced canvas, gloves, boots, and other gear parts [148]. Bulletproof vests, designed to absorb and halt projectile impacts, are generally classified into three types based on their structure and purpose: soft, complex, and multilayer. Polymer composites can be used in the manufacture of all three types [160]. The most common material combination for military protective equipment is ceramic, composite, and metal layers. Among composites, aramid fabrics such as Kevlar and Twaron, as well as ultra-high molecular weight polyethylene (UHMWPE), are widely used as layers in multilayer protective vests [161].

Research has demonstrated that hybridization significantly enhances the properties of kenaf/Kevlar/epoxy composites. Ballistic tests on these hybrid composites revealed that a composite with a weight ratio of 30/70 kenaf to Kevlar exhibits the highest ballistic protection and maximum energy absorption.

Polymer matrices reinforced with nanomaterials such as Kevlar and graphene enable the fabrication of exceptionally strong, lightweight, and innovative high-tech body armored vehicle. During the production of specific composites, thickening agents containing dispersions of nanoparticles—such as silicon dioxide nanoparticles in polyethylene glycol—are utilized. This process results in bulletproof vests that are more flexible, denser, and stronger. Consequently, soldiers benefit from enhanced mobility, improved protection against chemicals and toxins, and superior defense against firearms as well as blunt and sharp objects [156,157,158].

In the military industry, PBO fibers also have significant applications, primarily in ballistic protection, including body armor, helmets, and lightweight armored vehicles. Compared to aramids such as Kevlar, PBO fibers demonstrate superior mechanical resistance, reduced deformation upon impact, and improved thermal stability. A key requirement in this field is the efficient transfer of kinetic impact energy from the fiber to the matrix, which necessitates strong chemical and physical interfacial bonding. Surface modifications, such as treatments with methanesulfonic acid, alkaline activation, and plasma activation, significantly enhance the surface energy and polarity of the fibers. Composites with improved interfacial adhesion exhibit a transition toward cohesive failure modes and enhanced shock absorption capacity. The application of such modified PBO composites can be extended to lightweight combat vehicles, missile components, and tactical equipment. Research in this area may be directed toward maintaining ballistic performance after aging, exposure to moisture, and elevated temperatures [137].

Numerous studies have documented the advantages of graphene and nano-fillers in polymer composites for military applications [162,163,164].

### 4.4. Application of Polymer Composites in the Aviation Industry

The aviation industry is a major consumer of composite materials, accounting for more than 50% of the total annual composite production in the United States. The motivations for their extensive use are similar to those in the automotive sector, primarily focusing on reducing the weight of components, lowering production costs, and providing protection against various types of radiation.

Weight reduction of parts and the aircraft as a whole is critical as it influences key performance factors such as speed, fuel consumption, the complexity and number of manufactured components, maneuverability, and range [165].

The substitution of metal alloys with fiber-reinforced polymer composites enables cost savings by reducing the need for expensive production tools and decreasing the number of components, which in turn lowers expenses related to fasteners and simplifies maintenance procedures for parts and assemblies [159,166]. Moreover, this technology contributes to decreased environmental pollution.

Polymer composites also provide high protection efficiency due to the incorporation of fillers that exhibit resistance to X-rays and certain toxic substances [166]. For instance, silicone rubber is widely employed in aircraft construction owing to its excellent performance under extreme temperature conditions, resistance to various radiation types, chemical stability, ageing resistance, and outstanding electrical insulation properties. Additionally, carbon nanoparticles contribute superior oxidation resistance [167,168].

Given their specific mechanical, electrical, and tribological properties, polymer composites are well-suited for aerospace applications. These materials enable greater design flexibility for structures, power units, and a wide array of aircraft equipment. They also offer enhanced resistance to corrosion and wear, open flame and heat, improved damage and impact tolerance, endurance under various static and dynamic loads, noise reduction, vibration damping, and fracture resistance.

Consequently, polymer composites are employed in the manufacture of numerous aviation components, including aircraft brakes, compartment partitions, window frames, engine rotors, structural supports, fuselage sections, wing boxes, aircraft frames, turbine blades, vertical flaps, and tails of aircraft and helicopters [159,169].

The use of hybrid polymer composites reinforced with kenaf glass fibers increases the specific strength and resistance to rain erosion of aircraft surfaces. Additionally, silicon carbide polymer composites reinforced with carbon fibers, used in aircraft brakes, can withstand temperatures of approximately 1200 °C [121].

Numerous examples highlight the extensive use of polymer composites by leading aircraft manufacturers. Airbus, for instance, achieved a weight reduction of approximately 800 kg in its A320 model by substituting aluminum alloy components with polymer composites. This approach was similarly applied to the A330, A340, and A380 models [169]. Likewise, Boeing utilizes a significant amount of composite materials in its aircraft, with the Boeing 787 representing a notable breakthrough in the aviation industry [159]. Fiber-reinforced polymer composites contribute to reducing the fuel consumption of the Boeing 787 by over 20% [121,170].

A key objective within the aviation industry is to reduce carbon dioxide emissions, manufacturing costs, and fuel consumption. The use of bio-composites is increasingly contributing to these goals. Bio-composites are employed in the production of aircraft interior components, including seats, fairings, floors, and external body panels. These materials require high moisture and flame resistance as well as high specific strength for aircraft applications [171].

**Figure 10:**
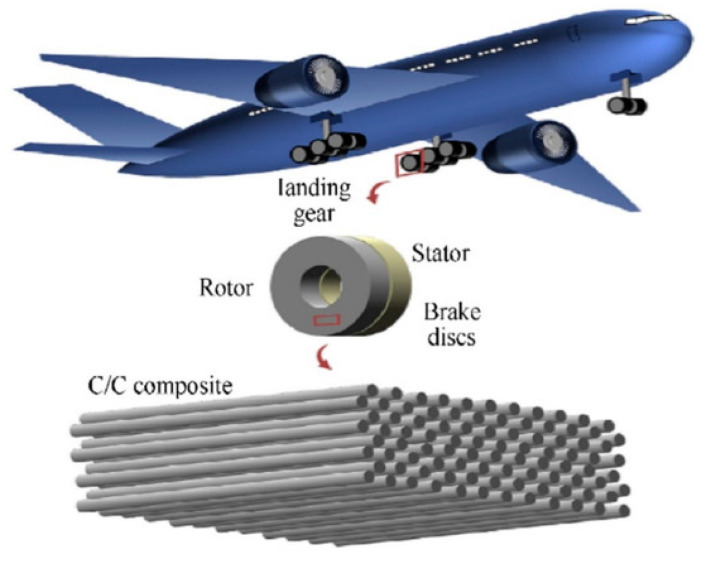
Schematic illustration of brake discs in the aircraft landing gears made with C/C material [174].

However, bio-composites generally exhibit low flame resistance, limiting their use in certain aircraft structures. Consequently, the external aircraft structure must be flame-resistant, while internal components such as cabins, decks, seats, and floors, which are less prone to fire risk, can be manufactured from bio-composites. A notable example is the production of interior cabin panels made from phenolic resin reinforced with woven linen [172]. The use of bio-composites has also been shown to reduce the weight of wing boxes by 12–14% compared to 7000 series aluminum alloys (Figure 10) [173,174].

The application of polymer composites in the aerospace industry is gaining increasing importance due to the demand for materials with high specific strength and stiffness, low weight, and excellent thermal and chemical stability. Poly (p-phenylene benzobisoxazole) (PBO) fibers represent one of the most promising materials in this field owing to their exceptional mechanical properties, such as high tensile strength, elastic modulus, and thermal stability. Moreover, PBO fibers exhibit flame retardancy and chemical resistance, making them suitable for extreme conditions in aerospace applications. However, one of the main challenges in using these fibers in composite systems is their inert and hydrophobic surface, which leads to poor adhesion between the fibers and the polymer matrix, thereby reducing the efficiency of stress transfer and the overall mechanical performance of the composites. Contemporary research is focused on surface modification of PBO fibers to enhance the fiber–matrix interface. Modifications such as plasma treatment, chemical oxidation, wet chemical treatment, and application of interfacial coatings have shown positive effects on adhesion without significantly compromising the inherent mechanical properties of the fibers. Particular attention is drawn to nanotechnologies, including functionalization with nanoparticles and nanostructured coatings, which enable precise control over the chemical and morphological characteristics of the surface. By improving fiber–matrix adhesion, the interlaminar strength of the composites is increased, fracture resistance is enhanced, and durability under operational conditions is improved. It is concluded that with appropriate surface modifications, the superior properties of PBO fibers can be fully exploited, making them highly attractive for use in primary and secondary aerospace structures where a combination of mechanical reliability, low weight, and resistance to extreme conditions is required. Continued development in surface chemistry and processing technologies will open new possibilities for integrating these high-performance composites into advanced aerospace systems [137].

## 5. Conclusions

The utilization of polymer composites for the production of various components, transport systems, protective equipment, and other applications across multiple fields—including medicine, industry, automotive, military, and aviation sectors—is of great importance.

Given that the automotive industry was one of the earliest adopters of polymer composites, this paper places particular emphasis on this sector, where their application remains most widespread. Due to their exceptional properties—such as high thermal conductivity or effective insulation depending on the composite type, low specific weight, excellent corrosion resistance, noise and vibration damping, design flexibility, and continuously decreasing production costs—polymer composites are increasingly replacing various metal alloys, steels of different grades, plastics, ceramics, and many natural materials.

Special attention should be dedicated to the development of methodologies and technologies that facilitate more efficient and simpler recycling of polymer composites. Similar to traditional metals, alloys, and other materials, recycling composites offers the advantages of lower cost and easier access to materials compared to newly produced ones.

Therefore, future research should focus on improving existing recycling methods and developing new approaches for composite materials. A crucial aspect of this research must be ensuring the safety of composite production, usage, end-of-life disposal, and recycling, both in terms of human health and environmental impact. This will guarantee that composites do not pose environmental risks and that their recycling aligns with sustainability principles.

Ultimately, enhancing the safety, efficiency, and environmental sustainability of polymer composites will be essential for their widespread future application, as well as for the advancement of new technologies that enable their sustainable use.

## Data Availability

The original contributions presented in this study are included in the article. Further inquiries can be directed to the corresponding author(s).
